# Yeast gene *KTI13* (alias *DPH8*) operates in the initiation step of diphthamide synthesis on elongation factor 2

**DOI:** 10.15698/mic2023.09.804

**Published:** 2023-08-08

**Authors:** Meike Arend, Koray Ütkür, Harmen Hawer, Klaus Mayer, Namit Ranjan, Lorenz Adrian, Ulrich Brinkmann, Raffael Schaffrath

**Affiliations:** 1 Institute of Biology, Division of Microbiology, University of Kassel, Heinrich-Plett-Str. 40, 34132 Kassel, Germany.; 2 Roche Pharma Research & Early Development, Large Molecule Research, Roche Innovation Center München, Nonnenwald 2, 82377 Penzberg, Germany.; 3 Max-Planck-Institute for Biophysical Chemistry, Am Fassberg 11, 37077 Göttingen, Germany.; 4 Environmental Biotechnology, Helmholtz Centre for Environmental Research - UFZ, 04318 Leipzig, Germany.

**Keywords:** budding yeast, EF2 diphthamide modification, diphtheria toxin, tRNA modification, elongator, tRNase zymocin

## Abstract

In yeast, Elongator-dependent tRNA modifications are regulated by the Kti11•Kti13 dimer and hijacked for cell killing by zymocin, a tRNase ribotoxin. Kti11 (alias Dph3) also controls modification of elongation factor 2 (EF2) with diphthamide, the target for lethal ADP-ribosylation by diphtheria toxin (DT). Diphthamide formation on EF2 involves four biosynthetic steps encoded by the *DPH1-DPH7* network and an ill-defined *KTI13* function. On further examining the latter gene in yeast, we found that *kti13*Δ null-mutants maintain unmodified EF2 able to escape ADP-ribosylation by DT and to survive EF2 inhibition by sordarin, a diphthamide-dependent antifungal. Consistently, mass spectrometry shows *kti13*Δ cells are blocked in proper formation of amino-carboxyl-propyl-EF2, the first diphthamide pathway intermediate. Thus, apart from their common function in tRNA modification, both Kti11/Dph3 and Kti13 share roles in the initiation step of EF2 modification. We suggest an alias *KTI13/DPH8* nomenclature indicating dual-functionality analogous to *KTI11/DPH3*.

## INTRODUCTION

Zymocin is a trimeric (αβγ) chitinase and tRNase toxin complex from *Kluyveromyes lactis* that kills *Saccharomyces cerevisiae* cells [1,2]. Expression in *S. cerevisiae* of its tRNase subunit γ alone (aka γ-toxin) is lethal [3] suggesting subunits α and β mediate zymocin contact with sensitive cells for γ-toxin uptake [2,3]. Accordingly, screens for zymocin survivors identified mutations in non-target (class I) and toxin-target (class II) genes termed *KTI* (*K**. lactis* toxin insensitive) [4]. While class I loci encode cell wall and membrane components (chitin, sphingolipids, H^+^ pump Pma1) for zymocin docking [5–7], class II genes identified the γ-toxin effector role of the tRNA modifier complex Elongator (Elp1-Elp6) [2,8,9]. Its tRNA acetylase subunit (Elp3) uses iron-sulfur (FeS) and radical SAM (RS) cofactors to modify wobble uridines (U34) in tRNA anticodons [10–12]. This includes methoxy-carbonyl-methyl-thio-uridine (mcm^5^s^2^U34) groups, which are hijacked for anticodon cleavage by γ-toxin. Hence, Elongator mutants lacking the mcm^5^s^2^U34 groups resist the tRNase attack [13,14].

**Figure 1:**
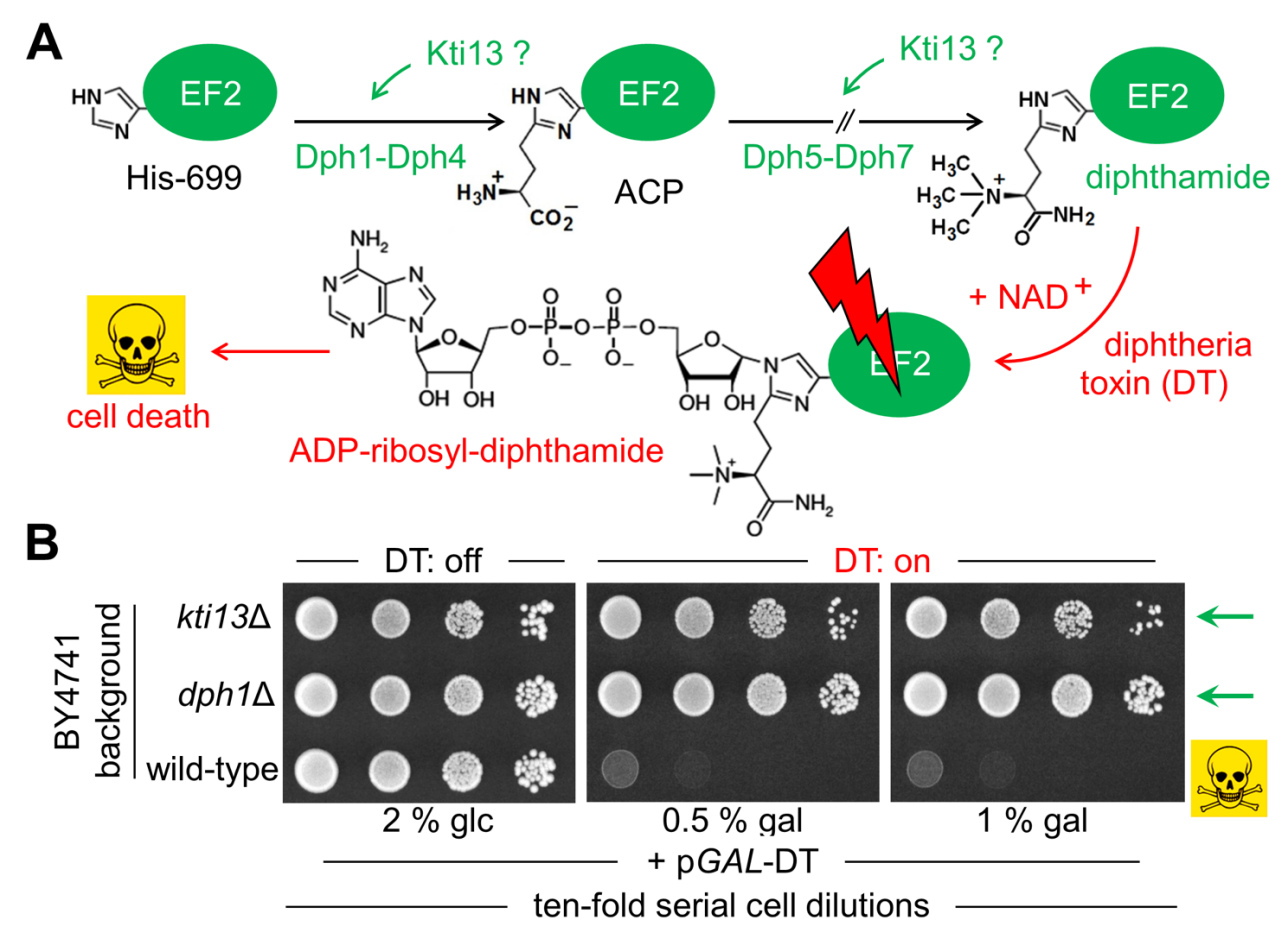
FIGURE 1: Potential role of yeast *KTI13* in diphthamide modification. **(A)** Simplified pathway overview [24,25]. Diphthamide synthesis initiates with modifcation of EF2 at His-699 by ACP involving proteins Dph1-Dph4. Subsequential reactions to convert ACP into end product diphthamide entail Dph5-Dph7. Potential Kti13 involvement in the synthesis steps is indicated (‘?’). Diphthamide can be hijacked by diphtheria toxin (DT) for ADP-ribosylation in an NAD^+^ fashion and induces cell death by EF2 inactivation (skull-crossbones). **(B)** *KTI13* and *DPH1* gene deletion strains resist against DT cytotoxicity. Yeast strains carrying p*GAL*-DT [39], a plasmid for galactose-inducible expression of the lethal ADP-ribosylase domain from DT (see A) were spotted onto medium containing 0.5-1% (w/v) galactose (gal) or 2% (w/v) glucose (glc). Following DT induction, growth inhibition of diphthamide-proficient wild-type is distinguishable from DT resistance of diphthamide-deficient *dph1*Δ and *kti13*Δ mutants (green arrows).

Among *KTI* loci not coding for Elongator subunits are regulatory genes: *KTI11* (aka *DPH3*), *KTI12, KTI13* and *KTI14* (aka *HRR25*) [2,4,15]. Kti12 binds tRNA and supports Elongator phosphorylation by kinase Kti14 [16,17]. Together with Sit4, a phosphatase antagonistic to Kti14, the tRNA modification activity of Elongator likely is phosphoregulated [9,15,18]. Kti11 is a rubredoxin-like electron carrier and dimerizes with Kti13 to effect Elongator-dependent tRNA modifications [18,19]. *KTI11* is also allelic with *DPH3* [20] and acts in diphthamide decoration of translation elongation factor 2 (EF2), a protein essential for life [21–23]. Diphthamide synthesis involves four steps encoded by a network (*DPH1-DPH7*) that is conserved in eukaryotes [24,25]. The EF2 décor is important for reading frame maintenance during mRNA translation and accurate protein biosynthesis [26,27]. Imbalanced proteostasis as a result of diphthamide deficiency has been attributed to neuropathies and various types of cancer in humans [28]. Of note, diphthamide underlies the human diphtheria disease since it is targeted by corynebacterial diphtheria toxin (DT) for ADP-ribosylation and EF2 inactivation [29] (**Fig. 1A**).

In line with dual roles for tRNA and EF2 modification, Kti11/Dph3 co-purifies with Elongator, EF2 and Dph1•Dph2 [30]. The latter enzyme uses (similar to Elp3) FeS and SAM cofactors for RS chemistry and formation of 3-amino-3-carboxyl-propyl-EF2) (ACP: **Fig. 1A**), the first diphthamide pathway intermediate [31,32]. Dph3/Kti11 in a dimer with Kti13, donates electrons to the FeS clusters in Dph1•Dph2 and possibly, Elp3 [33–36], which likely enables proper FeS redox states for RS-based modification chemistry. Whether the dimer feeds into both RS enzymes [18] or limits electron flow to Elongator as suggested [19] is moot. That *KTI11/DPH3* and *KTI13* gene functions may be related to each modification pathway, is supported by reports showing that both loci genetically interact with the Elongator and EF2 networks [18,22,37,38]. In relation to Kti11/Dph3, however, the precise role of Kti13 and its position within the diphthamide pathway have been less clear. On further studying *KTI13* gene function, we found that *kti13*Δ mutants survive EF2 inhibition by sordarin, a diphthamide-dependent antifungal, and evade ADP-ribosylation of EF2 by DT. Consistently, *kti13*Δ cells are drastically reduced in ACP formation indicating that proper initiation of the EF2 décor depends on Kti13. This is similar to Kti11/Dph3, which is why we suggest an alias nomenclature: *KTI13/DPH8*.

## RESULTS AND DISCUSSION

### *kti13*Δ phenotypes diagnostic for a *bona fide* diphthamide defect

**Figure 2:**
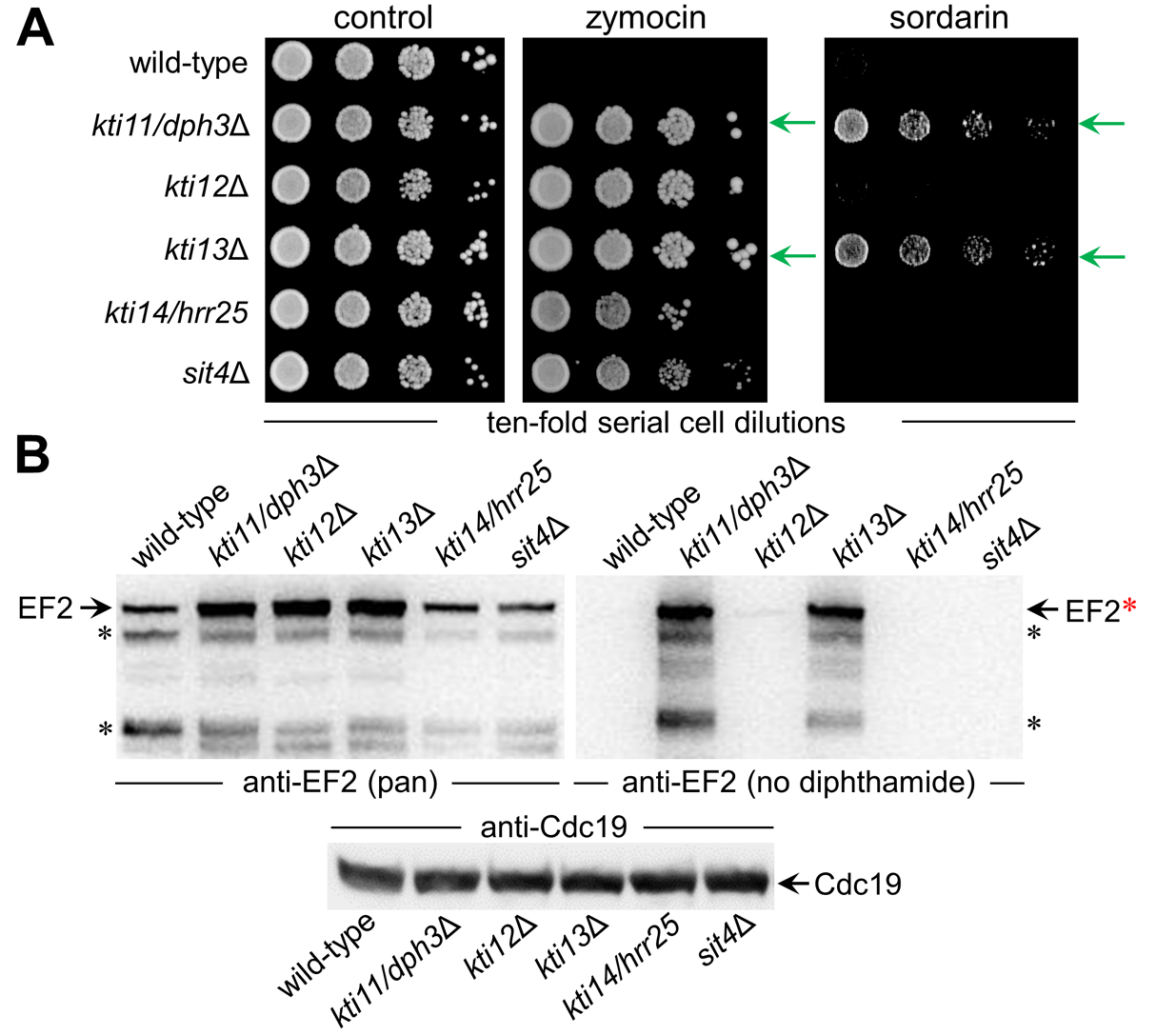
FIGURE 2: Among genes involved in Elongator regulation and tRNA modification, *KTI11* and *KTI13* also function in EF2 modification. **(A)** Growth assays in response to zymocin (0.02% [v/v]) or sordarin (9 µg/mL) and diagnostic for tRNA or diphthamide modifciation defects, respectively. Dilutions of cells with indicated genotypes were incubated at 30°C for 3 days. Note, that while all *kti*Δ and *sit4*Δ mutants resist growth inhibition by Elongator-dependent tRNase zymocin, only *kti11/dph3*Δ and *kti13*Δ cells are protected (green arrows) against diphthamide-dependent EF inhibitor sordarin. **(B)** Western blot analysis of total cell extracts from strains with genotypes as in A in order to profile their amounts of total EF2 and unmodified EF2 using anti-EF2(pan) (left panel) and anti-EF2(no diphthamide) antibodies (right panel), respectively. Black asterisks (left & right panels) denote EF2 degradation products, the red asterisk indicates full-length unmodified EF2 (right panel). The anti-Cdc19 antibody (bottom panel) was used as loading control. Note the anti-EF2(no diphthamide) Western blot (right panel) detects unmodified EF2 pools for *kti11/dph3*Δ and *kti13*Δ cells indicative for diphthamide defects.

To study Kti13 in more detail (**Fig. 1A**) we subjected a *kti13*Δ null-mutant raised in strain BY4741 to DT expression under *GAL-*promoter control [22,39]. In presence of galactose, DT expression was lethal to wild-type, while *kti13*Δ cells survived the toxin attack on EF2 (**Fig. 1B**). The resistance phenotype is robust and similar to the *dph1*Δ mutant (**Fig. 1B**), which is blocked in the first step of the diphthamide pathway [21]. Similar to other *kti* strains or mutants (*kti11/dph3*Δ, *kti12*Δ, *kti14/hrr25, sit4*Δ) lacking Elongator regulators crucial for tRNA modification [2,15,9,40], *kti13*Δ cells also copied zymocin resistance (**Fig. 2A**). When we compared growth of this mutant set in the presence of sordarin, a diphthamide-dependent EF2 inhibitor other than DT [22,41], solely *kti11/dph3*Δ and *kti13*Δ cells would protect against the antifungal (**Fig. 2A**). As shown previously, sordarin resistance is a trait diagnostic for failure to initiate or complete EF2 modification with diphthamide [39,41]. Thus, two out of five Elongator regulators tested, apparently share dual-functional roles in tRNA and EF2 modification pathways: Kti11/Dph3 and Kti13.

### An EF2 pool not modified with diphthamide accumulates in *kti13*Δ cells

Next, we analyzed protein extracts from the above set of mutants (*kti11/dph3*Δ, *kti12*Δ, *kti13*Δ, *kti14/hrr25, sit4*Δ) by Western blots (**Fig. 2B**). We used anti-EF2(pan), an antibody against EF2 regardless of modification, and anti-EF2(no diphthamide) shown to be specific for unmodified EF2 [29,42,43] (Fig. S1). *kti13*Δ cell extracts produced strong anti-EF2(no diphthamide) Western signals indicative for EF2 species not modified with diphthamide in absence of Kti13 (**Fig. 2B**). This is a read-out very similar to unmodified EF2 pools from *kti11/dph3*Δ cells (**Fig. 2B**), which like other step one mutants (*dph1*Δ, *dph2*Δ, *dph4*Δ) fail to initiate diphthamide synthesis (**Fig. 1A**) [20,21]. Previously, step one mutants were shown to raise EF2 protein levels, possibly to compensate for diminished EF2 function in absence of diphthamide [25,26]. Here, anti-EF2(pan) Western blots on *kti13*Δ and *kti11/dph3*Δ extracts also revealed upregulated EF2 levels (**Fig. 2B**). Thus, Kti13 and Kti11/Dph3 are diphthamide-related but differ from Kti12, Kti14 and Sit4, which are dispensable for making diphthamide based on anti-EF2(no diphthamide) blots (**Fig. 2B**). Nonetheless, we observed similar anti-EF2(pan) signals between *kti12*Δ and *kti13*Δ cells (**Fig. 2B**), suggesting an unheard EF2 upregulation under conditions that disturb tRNA (*kti12*Δ) but not diphthamide modification. In sum, among five known Elongator and tRNA modification regulators, two also contribute to diphthamide modification: Kti11/Dph3 and Kti13. Hence, to go with *KTI11/DPH3*, we suggest to SGD an alias nomenclature indicating bifunctional nature: *KTI13/DPH8*.

### *kti13*Δ cells block proper initiation of EF2 modification with diphtamide

**Figure 3:**
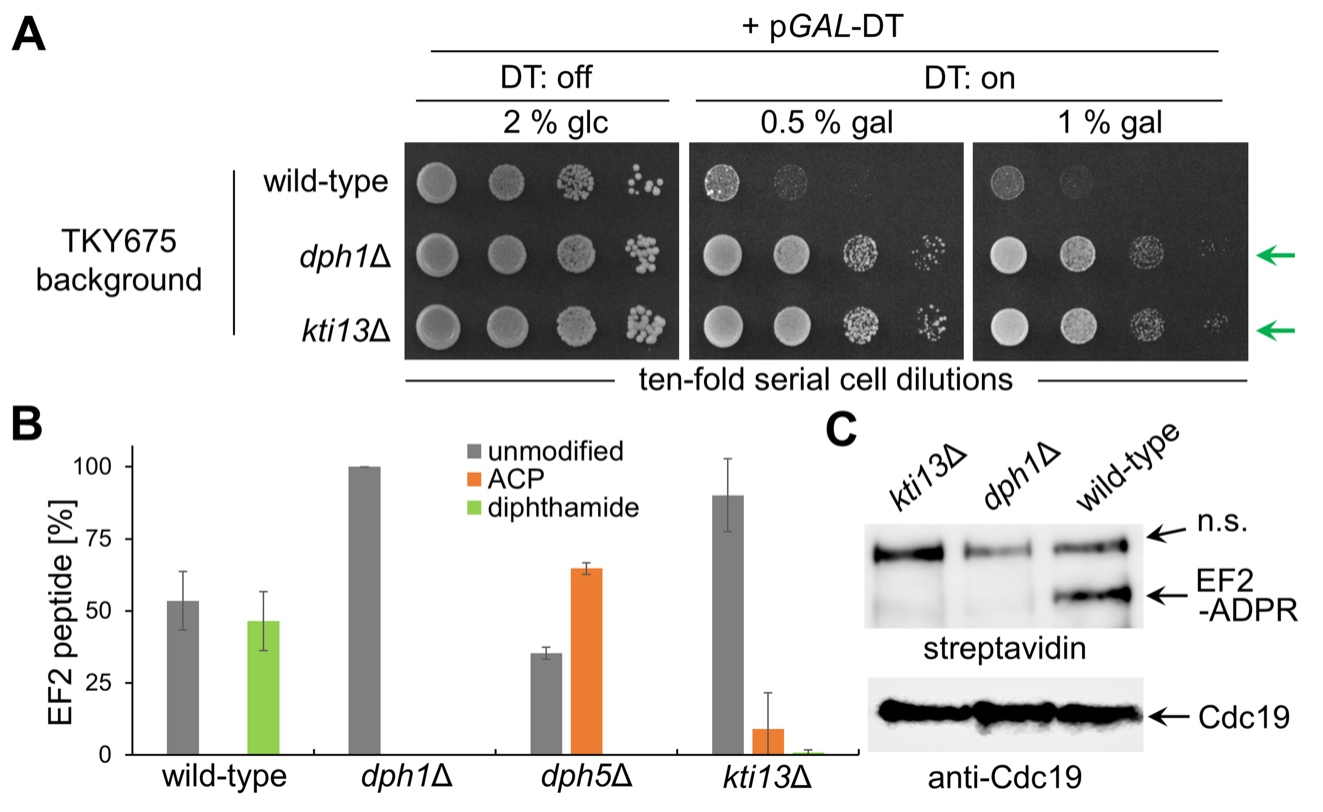
FIGURE 3: *KTI13* is required for proper initiation of diphthamide synthesis on EF2. **(A)** *kti13*Δ and *dph1*Δ mutants in strain TKY675 resist against DT cytotoxicity. The assay was essentially performed as for BY4741 (Fig. 1B). Following galactose-inducible DT expression, wild-type growth inhibition is distinct from DT resistance (green arrows) of diphthamide-deficient mutants (*kti13*Δ, *dph1*Δ). **(B)** Profiling diphthamide modification states on EF2 purified from wild-type, *dph1*Δ, *dph5*Δ and *kti13*Δ cells via nLC-MS/MS. Amounts of modification states were normalized to amounts of unmodified EF2 in *dph1*Δ (EF2 peptide [%]). *kti13*Δ contains pools of unmodified EF2 comparable to *dph1*Δ and drastically reduced ACP levels (∼9%) in relation to *dph5*Δ (∼65%). **(C)** ADP-ribosylation (ADPR) assay. Cell extracts from indicated genotypes were incubated with 200 ng DT and biotin-NAD [5 µM] at 25 °C for 1 h. The transfer to EF2 of biotin-ADP-ribose (EF2-ADPR) was detected by Western blot (top panel) using an HRP-streptavidin conjugate recognizing the biotin moiety of the reaction product [26,42]. An anti-Cdc19 Western blot (bottom panel) served as control for sample loading. Note that solely diphthamide-modified EF2 from wild-type cells undergoes detectable ADPR. As has been previously detected in similar assays [29,39], there is an unspecific (n.s.) reaction product of high molecular weight.

To further examine the position of Kti13 in the diphthamide pathway, we purified His-tagged EF2 from strain TKY675 [44]. Other than the full EF2 gene (*EFT1 EFT2*) complement of BY4741, TKY675 harbors a double knock-out (*eft1*Δ *eft2*Δ) with a single-copy plasmid carrying *EFT2-[His]*_*6*_ [44]. To diagnose diphthamide status in TKY675 prior to EF2 purification, we used DT assays (as above for BY4741). *dph1*Δ and *kti13/dph8*Δ mutants survived DT, yet their phenotype was weaker compared to BY4741 counterparts (**Fig. 1B**) and diminished by increasing DT loads (**Fig. 3A**). This suggests strain-specific variation due to EF2 copy number effects, a notion supported by Western blots showing significantly reduced EF2 pools and lower (than BY4741) levels of unmodified EF2 in *dph1*Δ from TKY675 (Fig. S1).

Next, we purified His-tagged EF2 from TKY675 in a two-step process coupling immobilized (IMAC: Fig. S2) with size exclusion chromatography (SEC: Fig. S3) for profiling diphthamide modification by nano-liquid chromatography tandem mass spectrometry (nLC-MS/MS) [43]. Previously, yeast, plant and human EF2 were found predominantly diphthamide modified [26,42,43]. In line with this scenario, we hardly detected any unmodified EF2 from total cell extracts of yeast strains BY4741 and TKY675 in anti-EF2(no diphthamide) Western blots (**Fig. 2B**, Fig. S1). However, upon purification of His-tagged EF2, nLC-MS/MS detected similar amounts of modified and unmodified peptides from TKY675 (wild-type: **Fig. 3B**). So, in contrast to normal *EFT1 EFT2* gene dosage and EF2 levels in BY4741, EF2 purified from TKY675 with single-copy *EFT2-[His]*_*6*_ apparently is not fully modified (Fig. S4). Whether this suggests the affinity-tag on EF2 or gene copy number reduction in TKY675 compromise the modification efficiency of the pathway is unclear. The observed imbalance, however, seems not to be accounted to the His-tag alone based on similar EF2 protein patterns in anti-His versus anti-EF2(pan) Western blots (Fig. S5).

Nonetheless, nLC-MS/MS on EF2 purified from the *dph1*Δ mutant reliably identified an unmodified tryptic peptide with no intermediate or modified variant detectable in TKY675 (**Fig. 3B**). This is consistent with earlier studies that exclusively identified unmodified EF2 in plant and human *dph1*Δ cell lines [42,43] and a yeast *dph2*Δ mutant lacking the Dph1 partner to initiate diphthamide synthesis on EF2 [39,45]. In control purifications from a *dph5*Δ mutant, which fails to use ACP (**Fig. 1A**) for formation of methyl-diphthine [39,46], we detected unmodified EF2 (∼35%) and ACP (∼65%) supporting previous data that ACP accumulates when step two of diphthamide pathway is blocked in the absence of Dph5 (**Fig. 1A**) [46,47]. Importantly, His-tagged EF2 purified from *kti13/dph8*Δ cells mostly appeared unmodified with minor ACP (∼9%) and low diphthamide (∼1%) amounts (**Fig. 3B**). Thus, nLC-MS/MS reveals similar profiles among *dph1*Δ and *kti13/dph8*Δ mutants strongly suggesting the latter has a step one defect and fails in proper formation of ACP-modified EF2, the first pathway intermediate (**Fig. 1A**).

### Unmodified EF2 from step one *kti13*Δ mutant escapes ADP-ribosylation by DT

In further support that *KTI13/DPH8* deletion copies diphthamide step one mutants are assays using biotinylated NAD^+^ as ADP-ribosyl donor [26,39] for ADP-ribosylation (ADPR) of EF2 by DT *in vitro*. Using an HRP-streptavidin conjugate to detect biotin in the ADPR reaction product [42], wild-type EF2 was found to yield robust bio-ADPR-EF2 signals (**Fig. 3C**). EF2 from *dph1*Δ or *kti13/dph8*Δ cells, however, lacked diphthamide-dependent ADPR acceptor activity indicating loss of diphthamide on EF2 evades ADPR by DT (**Fig. 3C**). These data fully agree with our anti-EF2(no diphthamide) blots showing unmodified EF2 from *kti13/dph8*Δ and *dph1*Δ (**Fig. 2B**; Fig. S1) cells and their DT resistance *in vivo* (**Fig. 1B**; **Fig. 3A**). These are features similar to *kti11/dph3*Δ cells lacking the electron donor that Kti13/Dph8 dimerizes with to drive Elongator-dependent tRNA modification [18,37]. Whether in analogy, the diphthamide function of Kti11/Dph3 also requires Kti13/Dph8 in the dimer for electron transfer and ACP synthesis by RS enzyme Dph1•Dph2 is plausible given drastically reduced ACP formation in *kti13*Δ cells (**Fig. 3B**) and *in vivo* traits typical of tRNA and EF2 modification loss caused by dimer interface mutations [18,22,37]. However, while electron transfer is essential for Dph1•Dph2 to form ACP *in vitro*, Kti13/Dph8 is dispensable in theses reconstitution assays [34,37].

## CONCLUSION

**Figure 4:**
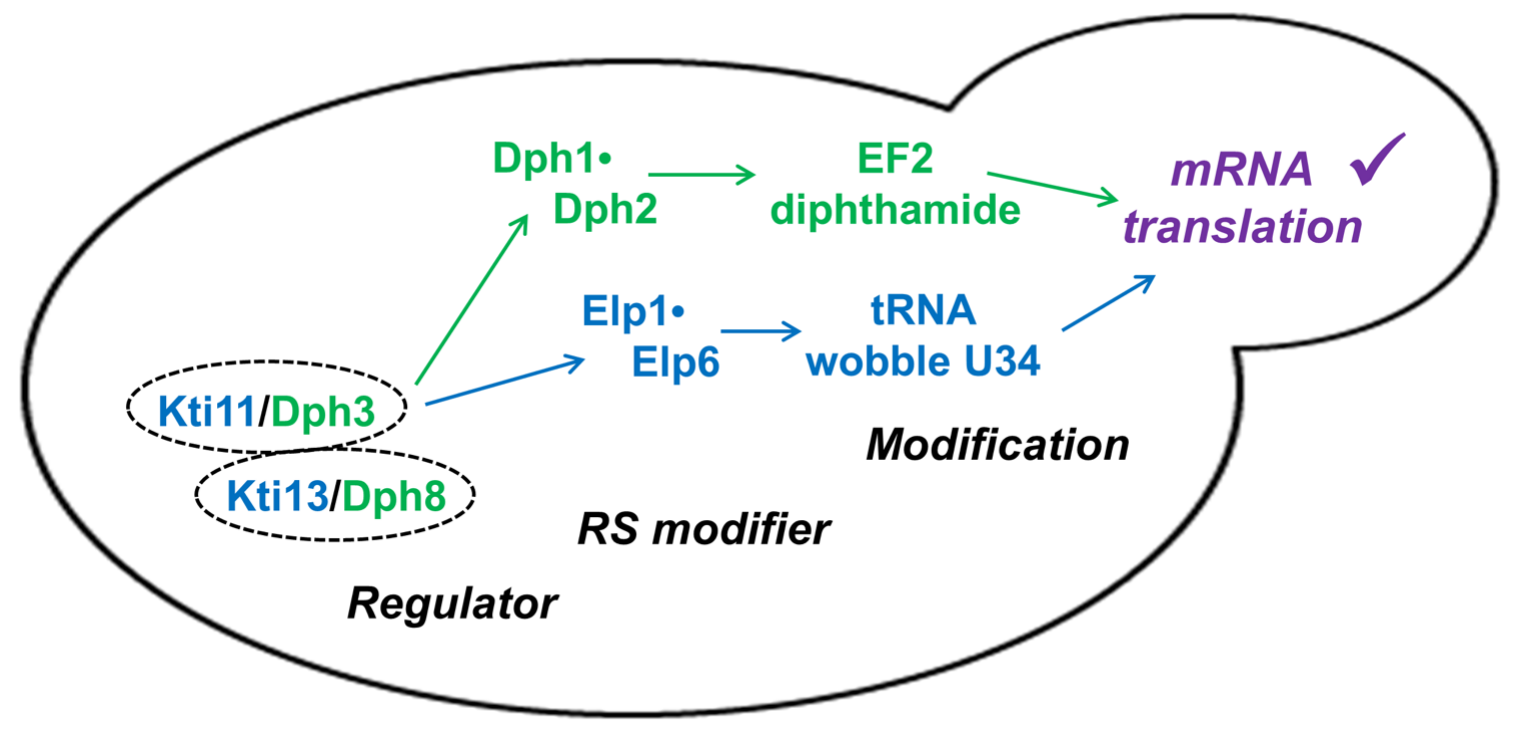
FIGURE 4: Kti11•Kti13 dimer (alias Dph3•Dph8), dual modification regulator. The dimer is located upstream of two radical SAM (RS) enzyme complexes (Dph1•Dph2; Elongator: Elp1•Elp6). Its dual regulator roles ensure proper synthesis of diphthamide on EF2 and modification of tRNA anticodon wobble uridine (U34) bases in order to support accurate mRNA translation and *de novo* protein synthesis [27,50].

We show here that apart from its effector role for Elongator-dependent tRNA modification in yeast, Kti13 alias Dph8 also operates in step one of the diphthamide modification pathway (**Fig. 4**). Although Kti13/Dph8 is important *in vivo* for EF2 modification by diphthamide, low ACP levels (∼9%) detectable in *kti13/dph8*Δ cells by MS suggest its presence for the diphthamide pathway to operate is not as catalytically critical as its partner protein Kti11/Dph3 [21,39]. In line with this, previous surveys on the tRNA modification pathway revealed low levels of Elongator activity (∼15%) in *kti13*Δ but none at all in *kti11*Δ mutants [9,40]. So residual EF2 and tRNA modification activity in the absence of *KTI13/DPH8* suggests an accessory role for gene product Kti13/Dph8. Perhaps it mediates proper electron flow from Kti11/Dph3 to either RS client (**Fig. 4**) for physiological modification reactions by Dph1•Dph2 and Elongator and thus, helps avoid inappropriate, harmful ones. Alternatively, in the dimer, Kti13/Dph8 may protect its RS clients against oxygen toxicity and FeS cluster damage as recently suggested for yeast and human Dph1•Dph2 [48,49]. Being located upstream of two RS modifiers (**Fig. 4**) that impact on the accuracy of tRNA decoding and EF2 translocation, a better understanding of how the Kti11•Kti13 (alias Dph3•Dph8) dimer affects mRNA translation is in need [27,50], particularly, in the light of clinically relevant roles for tRNA and EF2 modifications that have recently been shown to emerge in human disease syndromes [51,52].

## MATERIALS AND METHODS

### Strains, media, growth conditions and assays

*S*. *cerevisiae* and *K*. *lactis* yeast strains used throughout this study are listed in Table S1. Yeast gene deletion strains were generated based on PCR-mediated protocols using oligonucleotides and gene specific primers (Table S2) with pUG6 plasmid templates [26,39,53]. Strains were grown in complete yeast peptone dextrose (YPD) or minimal synthetic defined (SD) media [54] at 30°C unless otherwise stated. For zymocin response analyses, ten-fold serial cell dilutions of *S. cerevisiae* tester strains (starting OD_600_: 1.5) were spotted onto YPD plates lacking or containing 0.02-0.5% (v/v) zymocin. The latter tRNase complex was partially purified from *K. lactis* killer strain AWJ137 (Table S1) by ultrafiltration [55]. Incubation was for 2-4 days at 30°C. For sordarin assays, yeast cells were cultivated at 30 °C on YPD supplemented with 5-10 μg/mL sordarin produced from *Sordaria araneosa* (Sigma-Aldrich). DT growth assays involved galactose-inducible expression of the cytotoxic ADP-ribosylase fragment A [29] from DT, using vector pSU9 [39].

### Analysis of EF2 diphthamide modification status

Diagnosis of EF2 diphthamide modification states *in vivo* involved Western blots on total yeast cell extracts and antibodies that detect global EF2 pools irrespective of diphthamide modification (anti-EF2[pan]) or specifically recognize unmodified forms of EF2 (anti-EF2[no diphthamide]) [42]. Both antibodies were previously shown to detect human EF2 [42]. As the diphthamide target sequences between human (708-TLHADAIHRGGGQIIPT-724) [42] and yeast (692-TLHADAIHRGGGQIIPT-708) cells are identical [26], anti-EF2(no diphthamide) is suited to differentiate diphthamide modification states of EF2 from *S. cerevisiae* [26]. Total yeast cell extracts were generated as previously described [56] and protein concentrations determined by the Bradford assay [57]. 8 μl Lämmli samples were subjected to SDS-PAGE (12% [w/v] polyacrylamide) and blotted onto PVDF membranes (Merck/Millipore). These were probed overnight at 4°C with the anti-EF2(pan) anti-EF2(no diphthamide) antibodies [26] and developed with anti-rabbit secondary antibody HRP-conjugate (Dako; working concentration: 1:2000) and Lumi-Light Western blotting substrate (Roche) as previously described [26,42]. Protein loading was controlled in parallel Western blots with anti-Cdc19 antibodies recognizing pyruvate kinase. Diphthamide-dependent ADPR acceptor activity of EF2 in the presence of DT was tested *in vitro* [58]. The assays used total yeast extracts and biotinylated NAD^+^ as ADP-ribosyl donor for DT essentially as previously described with human and yeast EF2 resources [58,59].

### Two-step purification of His-affinity tagged EF2 by IMAC and SEC

His-tagged EF2 from strain TKY675 carrying *EFT2-[His]*_*6*_ on pTKB612 (Table S1) was detected with anti-(His)_6_ antibodies (Santa Cruz Biotechnology, USA) in Western blots. Purification by IMAC and SEC used 5 ml HisTrap columns (GE Healthcare, Chicago, USA) (Fig. S2, S3). For detailed IMAC and SEC protocols including modifications from the one originally described [44], see Supplemental Material.

### Detection of EF2 diphthamide modification states by mass spectrometry

Isolated EF2 proteins from the various TKY675 backgrounds (wild-type, *dph1Δ, dph5Δ* and *kti13/dph8Δ*) were analyzed via nLC-MS/MS to determine their diphthamide modification states in accordance with an earlier description for EF2 modification analysis from *Arabidopsis thaliana* [43]. Yeast proteins were separated by SDS-PAGE (7.5% [w/v] polyacrylamide), stained with Coomassie Blue, and excised as bands from the gel. Disulfides were reduced with dithionite and cysteine residues were alkylated with iodoacetamide, followed by trypsin digestion of proteins overnight, all within the gel piece as described [60]. Trypsin-digested fragments were eluted from the gel pieces and desalted using ZipTips [61] before analysis by nLC-MS/MS on a Thermo Orbitrap Fusion mass spectrometer (ThermoFisher) with injection via an electrospray ion source (Tri-Versa NanoMate, Advion). Acquisition of mass spectra was done at a resolution of 120,000 for MS1 scans and 60,000 for MS2 scans with operation parameters described in detail elsewhere [61]. Diphthamide-modified (C_81_H_137_N_25_O_23_), ACP-modified (C_78_H_130_N_24_O_24_) and diphthamide-unmodified (C_74_H_123_N_23_O_22_) precursor masses of 1829,03, 1786,97, and 1685,92, respectively, of target peptide 686-VNILDVTLHADAIHR-700 (with diphthamide target residue, His-699, underlined) were identified with ProteomeDiscoverer Version 2.4 (ThermoFisher) using SequestHT as the search engine and yeast EF2 (Eft1, Eft2) sequence as a database. Parameters included carbamidomethylation of cysteine as a fixed and the diphthamide modification of histidine (+C_7_H_14_N_2_O, m = 142.11 g) as a variable modification. We allowed no missed cleavage, a precursor charge state of +2 to +7, a precursor m/z tolerance of ±3 ppm, and a fragment mass tolerance of 0.1 Da. The false discovery rate was set to 1% at the peptide identification level using the Target Decoy PSM Validator node. Precursor abundance was estimated with the Minora node in ProteomeExplorer.

## SUPPLEMENTAL MATERIAL

Click here for supplemental data file.

All supplemental data for this article are available online at www.microbialcell.com/researcharticles/2023a-arend-microbial-cell/.

## Abbreviations:

KTI: – Kluyveromyes lactis toxin insensitive,
FeS: – iron-sulfur,
mcm^5^s^2^U34: – methoxy-carbonyl-methyl-thio-uridine,
EF2: – elongation factor 2,
DT: – diphtheria toxin,
nLC-MS/MS: – nano-liquid chromatography tandem mass spectrometry,
YPD: – yeast peptone dextrose.
