# Supplementary material for: Yeast gene KTI13 (alias DPH8) operates in the initiation step of diphthamide synthesis on elongation factor 2
Source: Microb Cell. 2023 Aug 8;10(9):195–203. doi: 10.15698/mic2023.09.804 (PMC10468694; doi:10.15698/mic2023.09.804)
Supplement: Supplementary file 1 [file mic-10-195-s01.pdf]

SUPPLEMENTAL MATERIAL

Yeast gene *KTI13* (alias *DPH8*) operates in the initiation step of diphthamide synthesis on elongation factor 2

Meike Arend[1], Koray Ütkür[1], Harmen Hawer[1], Klaus Mayer[2], Namit Ranjan[3], Lorenz Adrian[4], Ulrich Brinkmann[2] and Raffael Schaffrath[1,*]

[1] Institute of Biology, Division of Microbiology, University of Kassel, Heinrich-Plett-Str. 40, 34132 Kassel, Germany

[2] Roche Pharma Research & Early Development, Large Molecule Research, Roche Innovation Center München, Nonnenwald 2, 82377 Penzberg, Germany

[3] Max-Planck-Institute for Biophysical Chemistry, Am Fassberg 11, 37077 Göttingen, Germany

[4] Environmental Biotechnology, Helmholtz Centre for Environmental Research - UFZ, 04318 Leipzig, Germany

* Corresponding author: eMail: schaffrath@uni-kassel.de
Fon: +49-561-804-4175
FAX: +49-561-804-4337

1. Supplemental Tables

Table S1. Yeast strains used and generated in this study.

Strain	*Genotype*	*Source/reference*
Saccharomyces cerevisiae		
BY4741	*MAT***a** *his3Δ1 leu2Δ0 met15Δ0 ura3Δ0*	Euroscarf *
Y02262	BY4741 but *dph1Δ::kanMX4*	Euroscarf
Y00388	BY4741 but *kti13/dph8Δ::kanMX4*	Euroscarf
CBY12	BY4741 but *kti11/dph3Δ::kanMX4*	[1]
Y04960	BY4741 but *kti12Δ::KanMX*	Euroscarf
LL20	*MATα leu2-3,112 his3-11,15*	[2]
ARB97	LL20 but *kti4-1/hrr25-3*	[2]
Y03744	BY4741 but *sit4Δ::kanMX4*	Euroscarf
TKY675	*MATa ade2 leu2 ura3 his3 trp1 eft1Δ::HIS3 eft2Δ::TRP1* + pTKB612 (*EFT2-[His]$_6$ LEU2* CEN)	[3]
TKY675 *dph1Δ*	TKY675 but *dph1Δ::kanMX4*	This study
TKY675 *dph5Δ*	TKY675 but *dph5Δ::kanMX4*	This study
TKY675 *dph8/kti13Δ*	TKY675 but *kti13/dph8Δ::kanMX4*	This study
Kluyverromyces lactis		
AWJ137	*MAT***a** *leu2 trp1* [k1$^+$ k2$^+$]	[4]

* http://www.euroscarf.de/index.php?name=News

Table S2. Primers used for PCR-based generation of gene deletion and verification.

Name	*Sequence (5' → 3')*	*Application*
*DPH1*koF	CGCCTTGCCTAGCCAACAACTTTGTCGGTAGGTCCAAATCCAGCTG AAGCTTCGTACGC	*DPH1* ko **
DPH1 koR	TCGCTCCTGCCTATCTTCGAGATCGAGAACTCCAATCCAGTTGTGG CATAGGCCACTAGTGGATCTG	*DPH1* ko
DPH5 koF	AACGAACAGGATATAGAGTGAATAAAGGACAGTGAGAAAACAGCT GAAGCTTCGTACGC	*DPH5* ko
DPH5 koR	CATTATAAAAAAGAAACTACACATGAGCGTGTGCATTACCTGCATA GGCCACTAGTGGATCTG	*DPH5* ko
DPH8/ KTI13 koF	CGCAAGTGATGGAATGTGATCATTAAAGGCTATAACAGGCTTGTAT CCAGCTGAAGCTTCGTACGC	*DPH8/ KTI13* ko
DPH8/ KTI13 koR	ATGGACATCTATGTATATGATAGTGGGTATATAGTTACTTATCAGGC ATAGGCCACTAGTGGATCTG	*DPH8/ KTI13* ko
DPH1-F	CCATCAGTTTCGACCTCTTG	ko ver ***
DPH1-R	TAGTCACCGGTTGGGCATAG	ko ver
DPH5-F	GAGGAGTTGGCTTTCTTCAG	ko ver
DPH5-R	AGCGGACATTGCCGTGTACC	ko ver
KTI13-F	AGTCGGGTGTCCACCAGTAG	ko ver
KTI13-R	AGGGATAACGTCAGTCGGAGTTC	ko ver
kanMX-R	TTGGCAACGCTACCTTTGCC	ko ver

Abbreviations: ** ko: gene knock-out; *** ko ver: gene knock-out verification

2. Supplemental Methods

Two-step purification of His-affinity tagged EF2 by IMAC and SEC

Purification of His-tagged EF2 from reporter strain TKY675 carrying *EFT2-[His]*$_6$ on single-copy plasmid pTKB612 as sole genetic EF2 source [3] and its variants, i.e., *dph1*Δ, *dph5*Δ and *kti13/dph8*Δ (Table S1) was achieved by immobilized metal ion affinity chromatography (IMAC) (Fig. S2). We used 5 ml HisTrap columns (HP His tag protein purification columns, GE Healthcare, Chicago, USA) that enable the [His]$_6$ affinity-tag on EF2 to bind to nickel ions coupled to a sepharose resin. The IMAC protocol together with modifications from the previously described one by Jørgensen *et al.* [3] was as follows. TKY675 cells were grown in YPD at 30°C and harvested at OD_{600} ~3. After centrifugation (5.000 rpm, 20 min, 4°C) in a JA-18.1 fixed-angle aluminum rotor (Beckman Coulter, Brea, USA), the cell pellet was suspended in 1 ml per 1 g cells buffer A (50 mM potassium phosphate, pH 8.0, 1 M KCl, 0.2 mM PMSF, 1% tween 20, 10 mM imidazole). Cells were frozen in liquid nitrogen, grinded and thawed in lysis buffer (10 mM HEPES, 10 mM KCl, 1.5 mM $MgCl_2$, 2 mM benzamidine, 0.5 mM PMSF) containing 1 tablet EDTA-free protease inhibitor complete (Roche, Basel, Switzerland). The lysate was adjusted to pH 7.7 and centrifuged (12.500 rpm, 20 min, 4°C) in a JA-30.50 Ti fixed-angle titanium rotor, (Beckman Coulter, Brea, USA). Afterwards, the supernatant was centrifuged (45.000 rpm, 1 h, 4°C) in an ultracentrifuge type 45 Ti fixed-angle titanium rotor (Beckman Coulter, Brea, USA). The resulting supernatant was filtered through glass fibre filters and two HisTrap 5 ml columns were used for affinity purification of His-tagged EF2-His on an ÄKTA™ protein purification system (GE Healthcare, Chicago, USA). The columns were equilibrated with 5 column volumes (CV) of purification buffer A before the sample was loaded (~150 ml). The columns were washed with 5 C V of buffer B (50 mM potassium

phosphate, pH 8.0, 1 M KCl, 0.2 mM PMSF, 1% tween 20, 20 mM imidazole), and the proteins were eluted with 3 CV of buffer C (50 mM potassium phosphate, pH 8.0, 1 M KCl, 0.2 mM PMSF, 1% tween 20, 500 mM imidazole).

The elution was collected in 2 ml fractions, which were then loaded onto 12% SDS-PAGE (Fig. S2). to identify the fractions containing His-tagged EF2. Size exclusion chromatography (SEC) was performed with a HiLoad 26/60 Superdex 200 column (GE Healthcare, Chicago, USA) (Fig. S3). The column was equilibrated with 350 ml buffer D (20 mM Tris-HCl, pH 7.5, 100 mM KCl, mM EDTA pH 8.0, 10% glycerol, 1 mM PMSF, 2 mM DTT). The sample was loaded and run with a flow rate of 1 ml/min, collecting 2 ml fraction. Fractions were analyzed by SDS-PAGE (Fig. S3) to identify the ones containing His-tagged EF2 and verified by Western blots using anti-His antibodies (Santa Cruz Biotechnology, Dallas, USA). Pooled EF2 fractions were concentrated using a 30 kDa cut-off concentrator to a concentration of ~8 mg/ml and stored at -80°C.

3. Supplemental References

1. Uthman S, Bär C, Scheidt V, Liu S, ten Have S, Giorgini F, Stark MJ, and Schaffrath R (**2013**). The amidation step of diphthamide biosynthesis in yeast requires *DPH6*, a gene identified through mining the *DPH1–DPH5* interaction network. **PLoS Genet** 9: e1003334. doi: 10.1371/journal.pgen.1003334

2. Butler AR, White JH, Folawiyo Y, Edlin A, Gardiner D, and Stark MJ (**1994**). Two *Saccharomyces cerevisiae* genes which control sensitivity to G1 arrest induced by *Kluyveromyces lactis* toxin. **Mol Cell Biol** 14: 6306–6316. doi: 10.1128/mcb.14.9.6306-6316.1994

3. Jørgensen R, Carr-Schmid A, Ortiz PA, Kinzy TG, and Andersen, GR (**2002**). Purification and crystallization of the yeast elongation factor eEF2. **Acta Crystallogr D Biol Crystallogr** 58: 712–715. doi: 10.1107/s0907444902003001

4. Kämper J, Esser K, Gunge N, and Meinhardt, F (**1991**). Hetereologous gene expression on the linear DNA killer plasmds from *Kluyveromyces lactis*. **Curr Genet** 19: 109–118. doi: 10.1007/BF00326291

4. Supplemental Figures

Fig. S1:

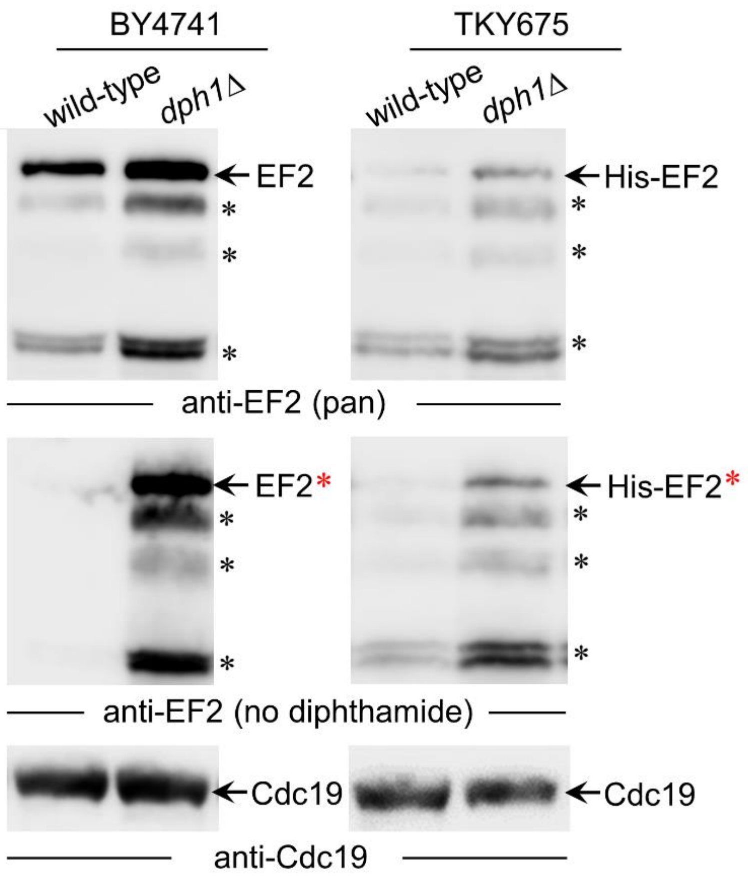

FIGURE S1: Western blots of total cell extracts from the indicated genetic backgrounds of strains BY4741 (left panels) and TKY675 (right panels). Amounts of total EF2 and unmodified EF2 were detected with anti-EF2(pan) (top panels) and anti-EF2(no diphthamide) antibodies (middle panels), respectively. The anti-Cdc19 antibody recognizing pyruvate kinase (bottom panels) was used as loading control. Black asterisks denote EF2 degradation products; detection of unmodified EF2 (middle panels) is indicated with a red asterisk. Note that in relation to BY4741, EF2 gene copy reduction of TKY675 [Table S1] causes lower-than-normal EF2 expression levels and that the *DPH1* gene deletion (*dph1*Δ) in either strain background triggers higher levels of unmodified EF2 (middle panels) compared to its wild-type parent.

Fig. S2:

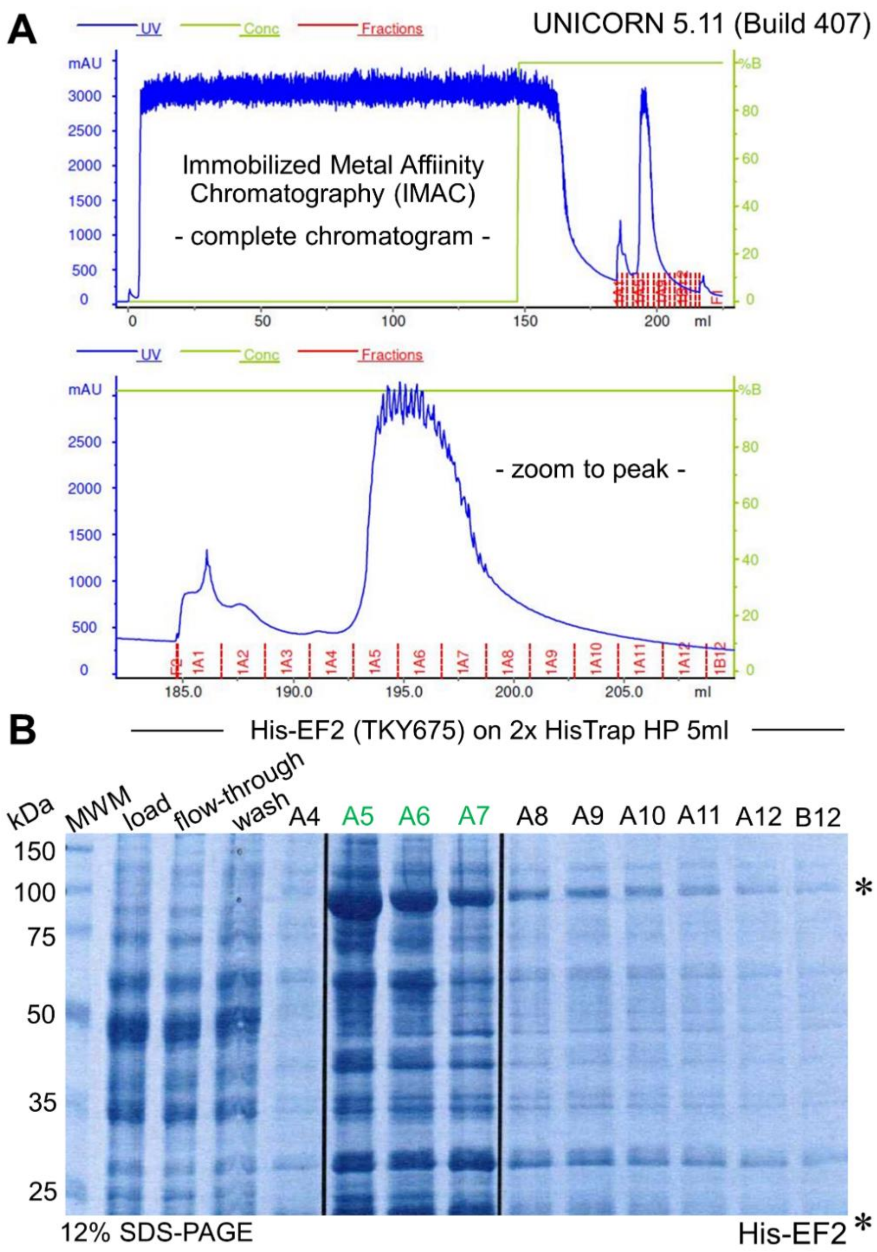

FIGURE S2: Purification of His-EF2 from TKY675 – IMAC. (A) IMAC was performed on an ÄKTA[TM] purification system using 5 ml HisTrap (HP His tag protein purification) columns (Supplemental Methods). Monitoring was done with the UNICORN 5.11 (Build 407) software. The report of the run is shown as complete chromatogram (top panel) or zoomed-in peak area (bottom panel). **(B)** SDS-PAGE and Coomassie stain to follow IMAC and purification of His-tagged EF2 at 95-100 kDa (marked by an asterisk). His-tagged EF2 was pooled (fractions A5-A7) for further purification by SEC (Fig. S3). MWM: molecular weight markers.

Fig. S3:

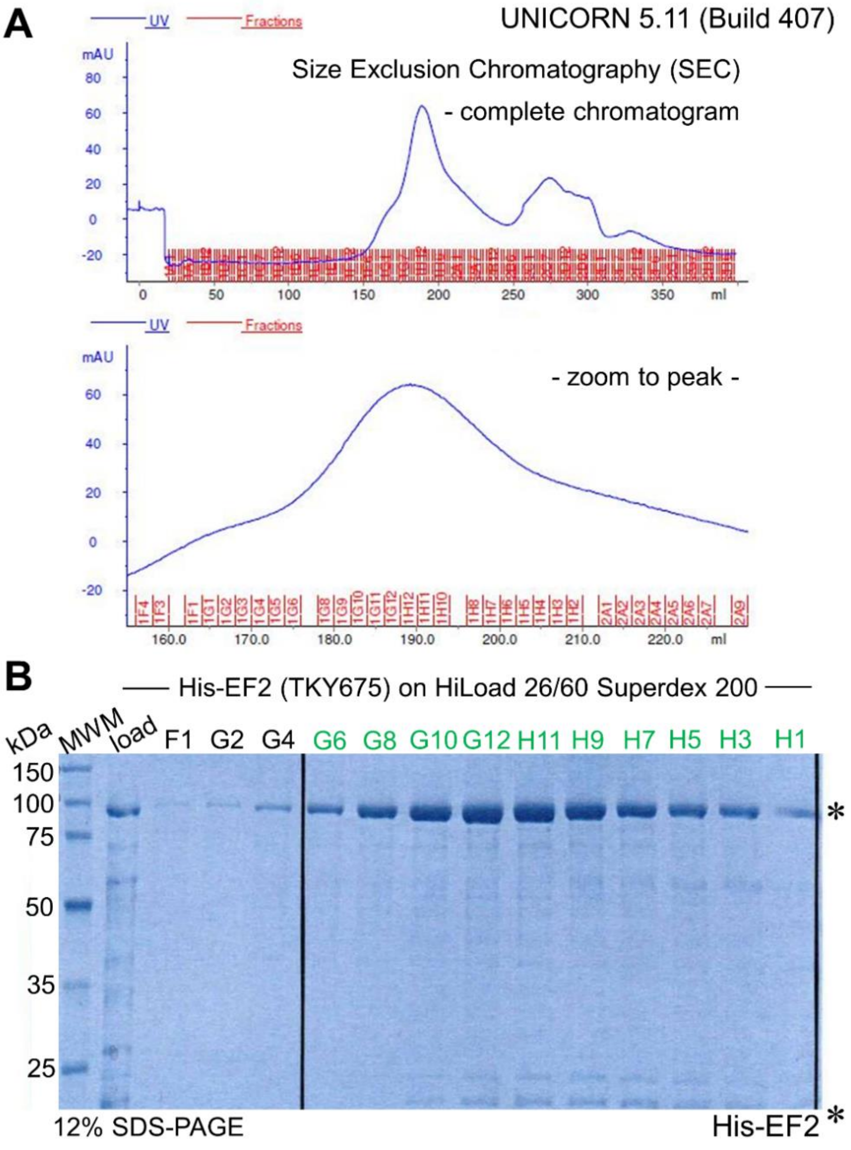

FIGURE S3: Purification of His-EF2 from TKY675 – SEC. (A) SEC was performed with a HiLoad 26/60 Superdex 200 column on an ÄKTA^{TM} system (Supplemental Methods, above) and monitored with the UNICORN 5.11 (Build 407) software. The run report of the complete chromatogram (top panel) and zoomed-in peak area (bottom panel) is shown. **(B)** SDS-PAGE showing EF2 purification upon SEC. The gel was stained with Coomassie Brilliant Blue. Purified His-tagged EF2 (marked by an asterisk) was pooled (fractions G6-G12 & H1-H11) and used for mass spectrometric analysis. MWM: molecular weight markers.

Fig. S4:

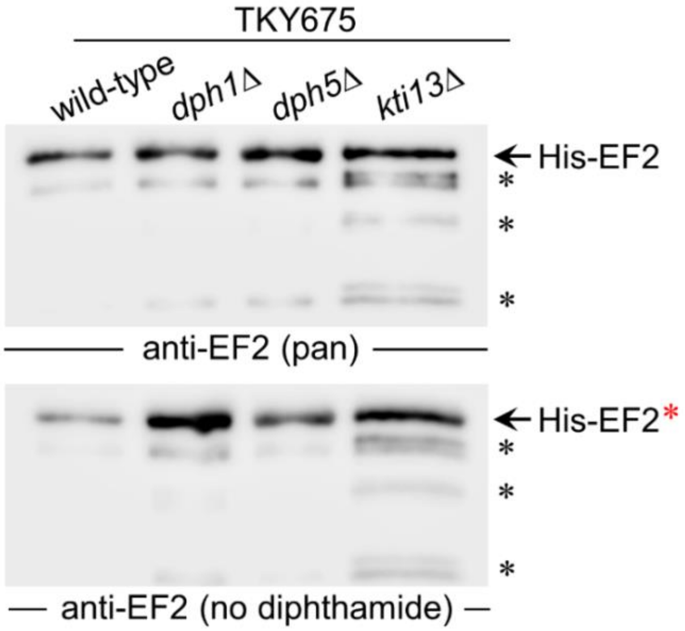

FIGURE S4: Analysis of diphthamide modifcation states on EF2 purified from the indicated TKY675 genotypes after IMAC and SEC. Total amounts of His-tagged EF2 and unmodified EF2 were detected with anti-EF2(pan) (top panel) and anti-EF2(no diphthamide) antibodies (bottom panel), respectively. A red asterisk indicates unmodified His-tagged EF2 (bottom panel); black asterisks denote EF2 degradation (both panels). Note that in relation to the TKY675 parent, higher levels of unmodified His-tagged EF2 purify from *dph1*Δ, *dph5*Δ and *kti13*Δ cells.

Fig. S5:

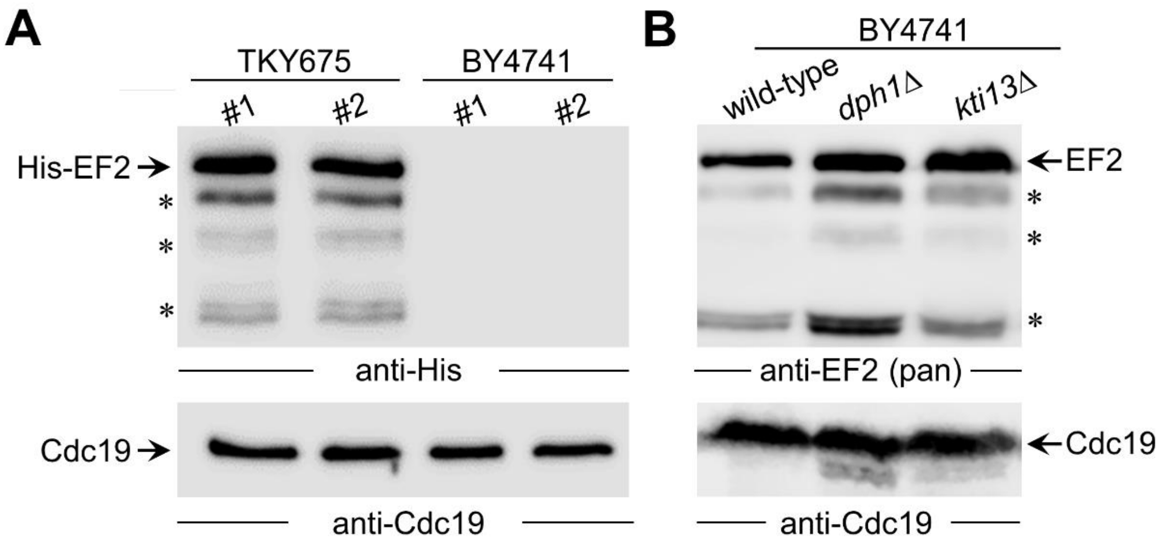

FIGURE S5: Comparative expression analysis between His-tagged and native EF2. (A) Western blot using anti-$(His)_6$ antibodies to confirm expression of His-tagged EF2 in cell extracts from two independent clones of TKY675 (*eft1*Δ *eft2*Δ p*EFT2-[His]*$_6$) [Table S1] not BY4741 (*EFT1 EFT2*). **(B)** Western blot using anti-EF2(pan) to follow up global EF2 levels in cell extracts from the indicated BY4741 genotypes. Asterisks denote EF2 degradation patterns similar between TKY675 and BY4741. An antibody recognizing pyruvate kinase (anti-Cdc19) was used as loading controls.
